# Unpredictable aggressive defence of the venomous snake, *Crotalus ravus*, towards predators and humans

**DOI:** 10.1242/bio.061791

**Published:** 2025-04-02

**Authors:** O. Azucena Núñez-Valdez, Melissa Plasman, Víctor Hugo Reynoso

**Affiliations:** ^1^ Departamento de Zoología/Pabellón Nacional de la Biodiversidad, Instituto de Biología, Universidad Nacional Autónoma de México, Circuito Exterior, Ciudad Universitaria, Coyoacán, Ciudad de México, C.P. 04510, México; ^2^ Centro Tlaxcala de Biología de la Conducta, Universidad Autónoma de Tlaxcala, Carretera Tlaxcala-Puebla km 1.5, Tlaxcala, C.P. 90062, México; ^3^ Dirección de Campos de Conocimiento y Desarrollo Docente, Universidad Rosario Castellanos, San Juan de Aragón II, Gustavo A. Madero, Ciudad de México, C.P. 07979, México

**Keywords:** Antipredation behaviour, Approach distance, Behavioural pattern, Defensive aggressive behaviour, Human health, Venomous bites

## Abstract

Antipredation behaviour is of high importance for the survival of prey animals, but it is also vital for the predator to understand the antipredator behaviour of potentially dangerous prey. Venomous snakes are particularly dangerous for their predators and humans, as a defensive bite may result in death. Here, we studied the behavioural response of the Mexican pigmy rattlesnake *Crotalus ravus* to the approach of simulated predators (birds and fox) and human, contrasting this to their predatory behaviour. Results showed that *C. ravus* defensive behaviour depended on the predator and was more aggressive towards humans. Mostly, for each type of behaviour the approach distance at first occurrence was similar among trials with different predators and reduced from freezing>rattling>escape>bite. However, we did not find clear behavioural patterns. In bird and fox trials, snakes always rattled or escaped before biting, however warning signals were not always displayed before biting and bite frequency was high in human trials, suggesting that this snake is dangerous for humans. Our results demonstrate that these snakes are flexible in their response to potential threats, but that the approach distance that elicits specific behaviours is mostly fixed.

## Teaser

**Summary:** Venomous snakes may respond with aggressive defence towards predators. Knowledge of their antipredation behaviour helps elucidate their decision-making during interspecific encounters, including with humans.

## INTRODUCTION

Interactions between predator and prey animals are important for the fitness of both species. For prey animals, antipredation behaviour is of high importance for their survival and most animal species have evolved a suite of antipredator behaviours and may decide which to use depending on the costs of the behaviour, the type of predator, and the risks involved (e.g. Greene, 1988; Lima and Dill, 1990; Eilam, 2005; Zhang et al., 2020). For example, many species present freezing to eliminate visual and auditory cues that the predator may use to detect the prey, during this behaviour the prey relies on its cryptic coloration (Eilam, 2005), but when the predator is closer or it is evident that it has seen the prey, the animal most likely flees (Edmunds, 1974; Martins, 1996; Eilam, 2005). Some animals also exhibit threatening displays and defensive aggressive behaviour (or aggressive antipredator behaviour; Eilam, 2005; Glaudas and Winne, 2007; Foster et al., 2015; Baxter-Gilbert et al., 2018; Horváth et al., 2020). Some prey species have evolved venom for use in defence, like bees, and some species use the venom that initially evolved to subdue their own prey in defence of potential predators, like scorpions or venomous snakes (Schendel et al., 2019).

Defensive agonistic behaviours are expected to be used only as a last resort (Whitaker et al., 2000; Eilam, 2005), when fleeing is not possible and other defensive threats are ineffective, as aggressive defensive behaviour implies energy costs and may include risks of injury as the predator is nearby (Abrahams and Dill, 1989; Lima and Dill, 1990; Preisser et al., 2005). However, defensive agonistic behaviours may lead to injury and even death of the predator (Mukherjee and Heithaus, 2013) making these behaviours beneficial, especially for strong or venomous prey species. Many dangerous prey species have evolved signals to repel potential predators before these dangerous interactions occur, either through aposematic coloration or threat display behaviour (e.g. gaping; Cuadrado et al., 2001; Glaudas and Winne, 2007; Mukherjee and Heithaus, 2013). Consequently, aggressive defensive behaviour may be avoided by the timely retreat of the predator when observing the threat displays. Nevertheless, high levels of threat may trigger a stronger defensive response (Helfman, 1989; Brown et al., 2006) and more dangerous predators could elicit agonistic responses instead of only threatening displays (Lima and Dill, 1990; Tozetti et al., 2009).

Venomous snakes may bite in their defence and are among the most dangerous animals for humans. Experts calculate that there are about 81,000 to 138,000 deaths and 2,700,000 cases of snake bite poisoning around the world every year (World Health Organization, 2021). The number of snake bites is rising, because anthropogenic activities cause habitat loss and bring snakes in close proximity to humans (Pitts et al., 2017; Glaudas, 2021; Goldstein et al., 2021). The potent and quick functioning poison in most snakes have caused an innate fear of snakes and each year humans kill millions of snakes (Wilson, 1993; Fernández-Badillo et al., 2021). Humans, then, form a strong threat for snakes and may provoke a strong defensive response. Snakes display a broad repertoire of anti-predation behaviours, related to the situation and the predator they confront (Greene, 1988). Many snakes display tail vibrations when threatened, probably to deter attention from the more vulnerable head (Greene, 1973, 1988). Rattlesnakes have likely evolved a rattle that currently functions as a warning signal to alert possible predators or humans of their presence (Greene, 1988; Allf et al., 2016; Reiserer and Schuett, 2016). Knowledge of the behaviour of snakes may foment appropriate response of humans to these behaviours and may aid in the prevention of ophidian accidents (Alves-Nunes et al., 2024).

In central Mexico, locals consider the Mexican pygmy rattlesnake *Crotalus ravus* very aggressive, and because of its high abundance in this densely populated area, this snake may cause many ophidian accidents in the surroundings of Mexico City (Fernández-Badillo et al., 2021; González Chávez et al., 2022). Here we investigated the antipredation behaviour of this snake to different types of predators (fox and bird) and its behaviours towards a prey and compared their actions with behaviours towards a human. Many snake attacks occur in humans ignorant of the presence of the snake (Coelho et al., 2019) and perhaps persons oblivious to the warning signals will perceive the venomous rattle snakes as dangerous, even when snakes generally only attack when harassed (Whitaker et al., 2000; Gibbons and Dorcas, 2002; Glaudas et al., 2005; Llewelyn et al., 2010). Our hypothesis was that snakes would only attack when the predator or human is very near, and rattling had not resulted in persuading to desist the approach of the possible predator. We predicted that the snakes would first freeze, then try to escape when the potential predator keeps approaching, then display threat signals (i.e. rattling), and only bite as a last resort when the potential predator is very close and that the order of these behaviours would not differ among predators. However, we predicted that snakes may act more aggressively towards a more dangerous predator (i.e. human), displaying warning behaviour at further distance and bite more frequently. Although aggressive antipredator behaviours may be modified predation behaviours (e.g. snake venom evolved for prey capture and then used for self-defence; Ward-Smith et al., 2020), strategies during predation avoidance likely differs from predation behaviour. We hypothesized that the strategies used during predation avoidance and possible human attack by the snake would differ from predatory behaviour, where we predicted only freezing and biting to occur.

## RESULTS

### Presence or absence of the behaviours

When rattlesnakes were approached by a human, an aerial predator (a bird silhouette overpassing and in a dive directly towards the snake) and a terrestrial predator (a stuffed fox), snakes responded with a range of behaviours: freezing, escape, threat displays (dorsoventral compression, rattling), and bite. They could also be observed elevating their head (an investigation behaviour) or hiding their head near or below the body (which may avoid damage in a possible attack; Arnold and Bennett, 1984). We considered bite, rattle, and escape *essential* antipredation behaviours, as the escape will reduce probability of confrontation, rattle will notify to an imminent confrontation, and with a bite ophidian accident may occur.

**Fig. 1.**
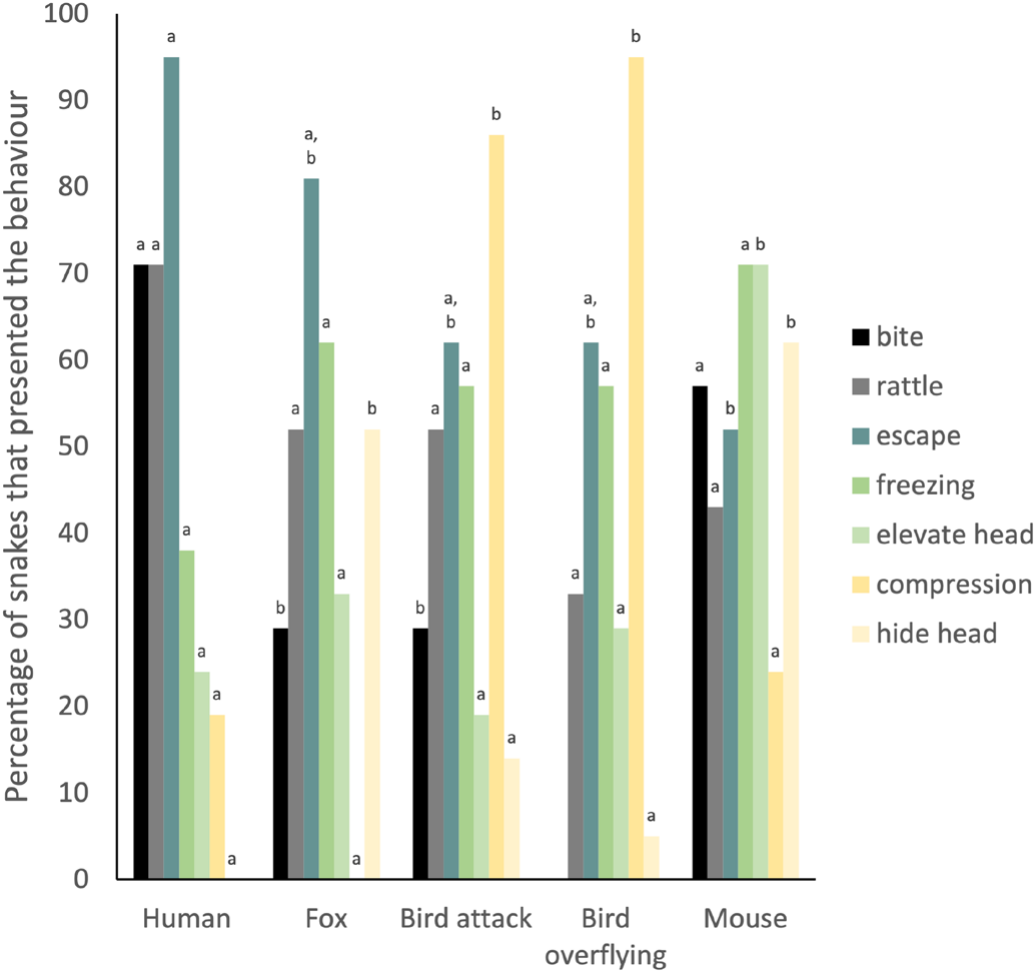
**Percentage of snakes that presented each behaviour in the tests.** Total number of snakes was 21. Letters indicate differences among the trials for the behaviour.

The presence of the behaviours during a trial, depended on the behaviour and the test (binomial GLMM: interaction: X^2^_24_=69.51, *P*=<0.001; AICc=826.88; Fig. 1; Table S1). Bite, rattle, and escape were displayed in high frequencies, especially in the test with humans. Some behaviours show similar frequencies among tests, like bite and rattle which have a frequency of 0.29 and 0.52 respectively in the tests with the attacking bird and the fox. An individual may have a consistent behavioural response in the different trials. For example, more aggressive individuals may respond with more agonistic behaviours to a threat (and are more likely to bite) than others. However, none of the individual snakes bit both in the trial with the fox and with the bird. Also, removing the identity of the snake improved the fit of the model (AICc=824.66). Hence, we did not find an effect of the individual in the response to different threats or evidence of consistent behavioural responses. The probability that a behaviour was present appeared to increase with environmental temperature (X^2^_1_=4.91, *P*=0.03); however, the effect size was small (slope=0.04). The time of the test did not affect the behaviour (X^2^_1_=0.1, *P*=0.09). Males and females did not present differences in their behavioural response (X^2^_1_=1.58, *P*=0.20).

### Initial behavioural response

**Fig. 2.**
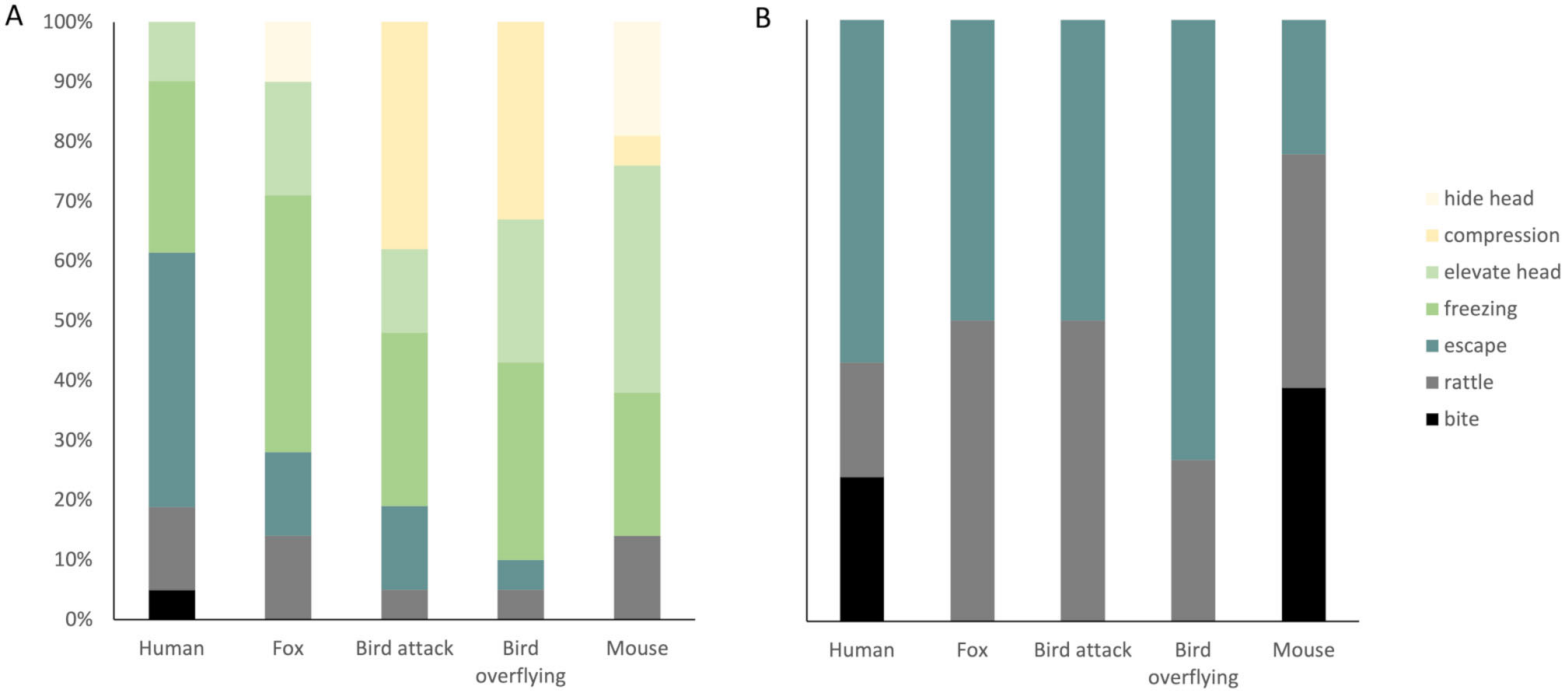
**The first behaviour displayed by the snakes in each test.** The percentage of snakes that displayed the behaviour in its first reaction is given for each test including all behaviours (A) or considering only the essential behaviours (B). Essential behaviours are bite, escape, and rattle, which are essential because these would cause (bite) or avoid (escape or warning through rattling) envenomation. *n*=21.

The initial behavioural response of the rattlesnakes depended on the predator stimulus (multinomial GLM; X^2^_24_=57.58, *P*<0.001; Fig. 2; Table S1). In the tests with birds, the first behaviour was most often the compression of the body, whereas the snakes first froze in the test with the fox, and they escaped more often from a human. In the presence of a prey animal (a mouse) the elevation of the head was mostly presented as the initial behaviour. Notably, only in the tests with humans, biting was observed as the first behavioural response, although just once. Temperature did not affect the initial behavioural response (X^2^_6_=7.54, *P*=0.27), nor did time (X^2^_6_=3.30, *P*=0.77) or sex (X^2^_6_= 0, *P*=1).

If we only consider the essential behaviours (i.e. bite, rattle, and escape), the first of these behaviours presented also depended on the test (X^2^_12_=35.96, *P*<0.001; Fig. 2B). In the test with the human and the overflying bird escape was generally the first essential behaviour, whereas in trials with the bird attack and fox rattling and escape were presented with similar frequency. In the test with the mouse, biting and rattling were equally frequent as the first essential response. Temperature (X^2^_3_=4.05, *P*=0.26), time (X^2^_3_=4.22, *P*=0.24), and sex (X^1^_6_=0.002, *P*=0.97) did not affect the first essential behaviour.

**Fig. 3.**
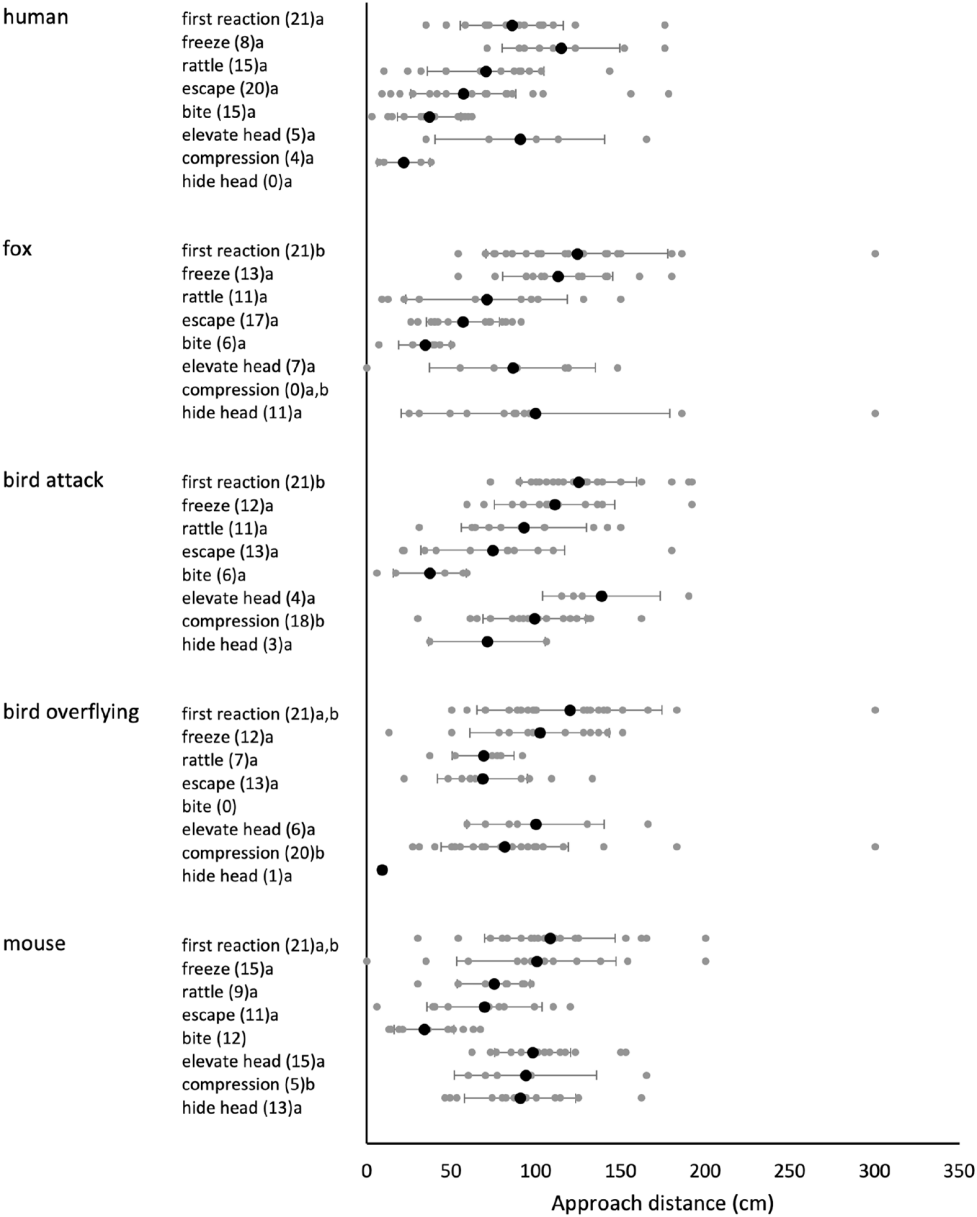
**Approach distance when behaviours are displayed for the first time by the snakes.** The approach distance is shown at the first occurrence of each behaviour in the tests. The black point represents the average, bars represent standard deviation, and the grey points indicate individual measures. The number of snakes that presented the behaviour is shown in brackets next to the behaviour and letters indicate differences among tests.

Interestingly, the distance at which the snake first reacted was shorter in trials with human (85.67±30.31 cm), than in the other trials (averages of 108 to 125 cm; Fig. 3; Tables S2 and S3). Reaction distance did not differ between the sexes (X^1^_4_=0.23, *P*=0.63). Among the different types of behaviours, only the approach distance at body compression differed among tests and was performed at a significantly shorter distance in the tests with the human (Fig. 3; Table S4), although at low frequency (Fig. 2). Approach distance at the expression of other behaviours did not differ among tests (Table S2). Approach distance did not affect flight distance (*F*_1,51_=2.51, *P*=0.12) or the rattle duration (*F*_1,23_=0.03, *P*=0.86). Sex did not affect approach distance (X^1^_4_=0.54, *P*=0.46).

The duration of rattling was significantly longer in the test with humans (36.67±28.59 s), compared to the tests with the birds and fox (averages of 6 to 13 s; Tables S2 and S5). Rattle duration in the mouse trial (24.11±13.09 s) did not significantly differ from that in the trials with humans and was significantly longer than in tests with the birds (Table S5).

### Behavioural sequence

Approach distance reduced from freezing to rattling to escape to bite. However, not all snakes presented all behaviours, and this complete sequence was only observed in four trials (which further also included other body postures) in different snakes and in different tests (once in the human test, once in the fox tests, and twice in tests with bird attack). Part of this sequence, freezing, rattle, escape, was observed three times, and freezing and rattle three times further. Contrarily to our prediction, the order of behaviours: freeze, escape, rattle, and bite, was only observed once, in a trial with a human and when omitting other body postures. This same order without the bite (i.e. freeze, escape, rattle) was only observed three times (once with a human, an overflying bird, and an attacking bird). Markov chain models (Tables S6-S8) show that there is no clear pattern in the order of the different behaviours. However, in the test with humans, bite was predicted to follow rattling, compression, and elevation of the head in half of the cases.

### Warning signals

**Fig. 4.**
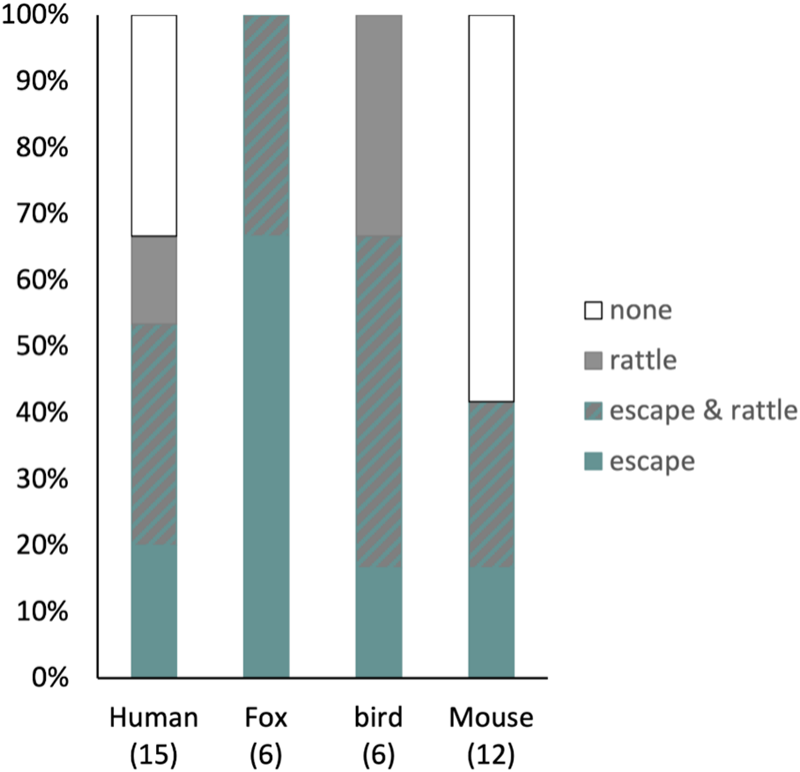
**Defensive behaviours presented before biting.** The percentage of the snakes that bit (number below the name of the test) that presented no defensive behaviour, that rattled, and/or escaped before biting in each test. For the test with the birds, only tests of the bird attack are shown, as in the test with the overflying bird bites never occurred.

Evaluation of the behaviours prior to the bite to show the danger of a rattle snake to a potential predator, showed that in the tests with humans a high number of snakes bit (15 of 21, or 71%), and only 67% of these snakes rattled or escaped before biting. Whereas in the tests with the bird attack and the fox, 29% of the snake bit (in both tests) and in all these cases the snake rattled and/or escaped prior to biting (Fig. 4). The fox and bird approached in a straight line to the original location of the snake, whereas the human kept approaching even when the snake fled, which could have led to higher aggression levels in these trials. However, in 33% of the trials with humans, the snakes struck before escaping and when biting occurred, 33% of the snakes bit before escaping or warning the potential predator by rattling (Fig. 4). Interestingly, the number of snakes that bit was lower in the tests with mice than in the test with humans; only 12 of 21 snakes bit the mice in the duration of the trial, and of these snakes 64% rattled or escaped (i.e. moved away) before biting.

## DISCUSSION

Our study evaluated the behaviour of the rattlesnake *C. ravus*, a venomous species considered aggressive by locals and of a high interest for public health. Our results confirm that, when approached, this species can be dangerous. Although most snakes initially responded to an approach with escape or freezing, the snakes struck in many trials (up to 71% in the tests with humans; Fig. 1) when the potential predator was near (approximately 35 cm; Fig. 3). More snakes bit in the trial with a human (sometimes even before escape or rattling), than in the trials with the fox or bird and even mice (Fig. 1). These results suggest that *C. ravus* may be more aggressive than other snakes, as in most other studies snakes only bit when touched or harassed (Whitaker et al., 2000; Gibbons and Dorcas, 2002; Glaudas et al., 2005; Llewelyn et al., 2010; but see Herr et al., 2017). Our study further showed that *C. ravus* displayed different behaviours depending on the predator and did not have a structured behavioural antipredation response, which makes these snakes unpredictable and therefore more dangerous.

Our results showed that *C. ravus* antipredation behaviour depended on the predator species. The benefits of certain defence behaviours may be context dependent (Bateman et al., 2014) and prey animals may respond according to the hunting strategies or the potential threat of the predator (e.g. Kavaliers and Choleris, 2001; Colombelli-Négrel et al., 2010; Lourenço-de-Moraes et al., 2016; Farrow et al., 2017), including rattle snakes (Scudder and Chiszar, 1977; Alves-Nunes et al., 2023). For example, animals may flee under bushes to prevent avian predation, whereas this may be less successful against terrestrial predators, which may even be luring there (Kotler et al., 1992).

We found that the snakes reacted more often to a bird by compressing their body, whereas compression was never observed at the approach of the fox, where snakes more often froze (Fig. 1). In many species, animals rely on camouflage when a predator approaches and freezing increases crypsis by reducing visual stimulation and thereby reduces the probability of perception by the predator. Pit vipers tended to display cryptic behaviours more often towards an aerial predator than a terrestrial predator, but also displayed more body compression towards aerial predators (Alves-Nunes et al., 2023). Cryptic behaviour is only useful when the prey has not been spotted, otherwise it becomes an easy target (Eilam, 2005; Lourenço-de-Moares et al., 2016). When the prey animal knows it has been perceived by the predator, it may respond with behaviours to dissuade the predator from attacking. Body compression may increase body size appearance and can be used as a threat signal (Arnold and Bennett, 1984). In tests with humans, compression was followed by either rattle or bite, suggesting that body compression may also be a threat signal in *C. ravus*. However, body compression was also observed in high frequency in the presence of prey animals (mice), and this body posture may perhaps foment crypsis (Webster et al., 2009). Although it is also possible that the novel stimulus represented by the mouse may have triggered a threat response.

Aggressive defensive behaviours can be risky (the predator is close by) and energetically costly (Abrahams and Dill, 1989; Lima and Dill, 1990; Preisser et al., 2005). It would be expected that prey animals only attack when they were unable to avoid detection and warning behaviour was ineffective to dissuade the predator (Whitaker et al., 2000; Eilam, 2005). Following the distance-dependent defence hierarchy (Gallup, 1974), approach distances decreased in the order of freezing-rattling-escape-bite, and were independent of predator species, excluding the approach distances of body postures, which differed among predator species or showed high variation (Fig. 3). This appears to suggest a structured hierarchic defence strategy during the approach of a predator of freezing to avoid detection, followed by threatening behaviour, then escape, and biting as a last resource. A hierarchy in the decisions of antipredation behaviour has been observed in other snakes, in which the snakes respond more strongly when the threat persists or when the snake is touched instead of only approached (e.g. Gibbons and Dorcas, 2002; Shine et al., 2002; Roth and Johnson, 2004; Cox et al., 2020). However, we did not find a clear sequence of behaviours (see the Markov models in the Tables S6-S8), as individual snakes did not display all behaviours within a test.

Importantly, snakes struck in many trials with humans and sometimes without previously escaping or show of threatening behaviour. Also in the venomous cottonmouth, *Agkistrodon piscivorus*, snakes may strike before showing threatening signals (Glaudas and Winne, 2007). Animals may opt for a more aggressive defence when perceived threat level is high (Eilam, 2005; Nisani and Hayes, 2010). For snakes, human encounters are often dangerous, frequently resulting in harm or death (Fernández-Badillo et al., 2021), and the snakes may perceive humans as an important threat. Aggression was higher towards the human even though these tests were performed in their natural environment with refuge possibilities, which may favour flight over aggressive defence (Whitaker and Shine, 1999; Eilam, 2005; Zani et al., 2009; Blanchard et al., 2011), compared to the barren environment in which the other tests took place. Yet snakes were never observed to enter a refuge. Refuge use may impose costs in thermoregulation (Cooper and Wilson, 2008) or loss of time for other important activities (Reaney, 2007) or a good refuge may not readily be available.

Alternatively, the snakes may not have perceived the models of the fox and the bird as dangerous. Crotaline snakes are very sensitive to thermal cues and may be more likely to strike warm objects (Safer and Grace, 2004), which could have led to a higher attack frequency in humans. Therefore, we warmed the fox prior to the tests, and even though the perception of this stimuli may differ from that of a real fox, 29% of the snakes bit. However, we did not monitor the temperature of the fox during the tests and behaviours towards the fox may have been affected by differences in temperature of the model approaching. In the test with the attacking bird also 29% of the snakes struck, and the bird silhouette used was at environmental temperature. It is possible that snakes may be visually oriented (Schraft and Clark, 2019) and rather than solely depending on thermal imaging, this technique may be used to sharpen the visual image (Hartline et al., 1978; Bakken and Krochmal, 2007). Many snake species show defensive antipredation behaviour towards unheated, inanimate objects (e.g. Glaudas, 2004; Whitford et al., 2020; Gibert et al., 2022).

Harassment may increase gaged threat level (Hayes et al., 2002) and may result in a biting probability of up to 100% in some species of rattlesnakes (Whitford et al., 2020), however the fox and bird approached the snake only once and in a direct line, and were easily avoided, bestowing thus minimal harassment, and therefore the behaviour of the snakes observed was probably a genuine antipredation response.

Our results showed that individual snakes responded differently to the stimuli. Behavioural differences observed among individuals may have been caused by intrinsic factors. Snake antipredation behaviour often varies with body size (e.g. Delaney, 2019; Whitford et al., 2020). The main purpose of this study was to evaluate the risk of ophidian accidents by this species, for which size of individuals would be irrelevant, since these snakes are always dangerous. However, we did not find an effect of the individual among the tests, indicating that body size did not affect the tests.

Behavioural variation among tests may have been affected by temperature. Body temperature often affects antipredator behaviours of snakes (Shine et al., 2002; Llewelyn et al., 2010; Whitford et al., 2020). Although we were not able to measure body temperature during the experiments, in ectotherms this is generally related to environmental temperature (e.g. Jaramillo-Alba et al., 2020). We found that environmental temperature had only a small positive effect on the probability of the presence of the behaviours and did not affect the initial response or approach distances.

Surprisingly, we observed rattling in the mice trials. Handling may cause stress (Balcombe et al., 2004; Schuett et al., 2004; Holding et al., 2014) which may deteriorate or inhibit foraging behaviour (e.g. Pankiw, 2003; DesRoches et al., 2022; Yang et al., 2022) and perhaps result in defensive behaviours to new stimuli. The mouse was placed in the arena shortly after the snake, and the handling may have stressed the snake. This may also explain the low frequency of predation (12 out of 21 snakes bit the mouse and only six snakes ate the mouse within the duration of the trial). Interestingly, snakes that ate the mouse never rattled. Perhaps only hungry snakes presented predation behaviour, and although snakes were fasted for five days prior to the trial this may not be enough to reach a hunger state as snakes can go without food for long periods (McCue, 2007). Snakes that are not hungry may respond to the mouse as to harassment, rattling can be observed in rattlesnakes that are harassed by adult squirrels that are defending their young (Swaisgood et al., 1999). Also, as wild animals, these snakes may not directly identify laboratory reared mice as prey or require a hiding place from which they can ambush (Chiszar et al., 1996; Clark, 2005). Nevertheless, all snakes finally ate the mice. Alternatively, the snake could be responding to a lingering presence of human odour either on the mouse or behind the fences. When perceived predation pressure is high, the animal may present antipredation behaviours in absence of the predator (Carr, 2002; Creel, 2018). However, in this case, the behaviour would be expected in all trials as models were handled by humans and a human was always nearby to observe the snakes.

Snakes bit at distances from 3 to 62 cm, comparable to bite distances of other *Crotalus* species (Clark et al., 2012; Whitaker et al., 2000). Interestingly, most snakes bite their prey in very close proximity (3-20 cm), whereas defensive strikes can be taken from up to 50 cm (Clark et al., 2012) or 60 cm (Whitaker et al., 2000; this study), but we found no difference in strike distance of defensive strikes to predatory strikes (Table S2).

The high speed of these defensive attacks (LaDuc, 2002; Penning et al., 2016) makes it impossible for humans to avoid the bite (Coelho et al., 2019). The threat may be less severe when animals bite without injecting venom (i.e. dry bite; reviewed in Naik, 2017), which in rattlesnakes may occur in 10-25% of bites in humans (Minton, 1987; Hayes et al., 2002). The startle effect from the bite may be sufficient to ward of the attack and venomous animals may invest less venom in defensive strikes than in predatory bites (Hayes et al., 2002; Nisani and Hayes, 2010). Here, we did not investigate the quantity of venom in a bite given and small quantities may be difficult to observe yet be lethal (Minton and Weinstein, 1984). However, when the perceived threat is high, animals are more likely to inject venom (Hayes et al., 2002; Nisani and Hayes, 2010) and some *Crotalus* species appear to invest more venom in defensive bites than predatory bites (Young and Zahn, 2001; Hayes et al., 2002). Still, the persistence of the predator stimuli may strengthen the stress reaction triggering a fight or flight response and short exposure may lead to less aggressive reactions (Buchanan, 2000).

We hoped that this study helped to offer strategies to avoid ophidian accidents and expected to find that all snakes would escape or rattle before striking. However, our results show that this is not the case. Moreover, we found that the snakes were especially aggressive towards humans. The small approach distance for biting and considering that most snakes flee or rattle before biting (67%), suggest that a vigilant person likely can avoid being bitten.

## MATERIALS AND METHODS

### Study organism

*Crotalus ravus* (Cope, 1865) is a rattlesnake that can reach a snout-vent length of 40-65 cm (Uribe-Peña et al., 1999; Campbell and Lamar, 2004), presents a cryptic coloration pattern of browns and greys (Campbell and Lamar, 2004), and feeds on insects, small mammals, and lizards (Díaz de la Vega-Pérez et al., 2016; Calzada-Arciniega et al., 2016). This snake is found in the highlands of central Mexico from 1490 to 3000 m above sea level, where it inhabits pine-oak forest, mesophilic mountain forest, low deciduous forest, tropical deciduous forest, mesquite, and xerophytic scrub (Uribe-Peña et al., 1999; Campbell and Lamar, 2004). *C. ravus* is listed as threatened according to the Mexican government (NOM-059-SEMARNAT, 2010). This study complies with the current regulations of Mexican law on animal welfare, and the project was carried out with permissions for scientific research from the Secretaría de Medio Ambiente y Recursos Naturales (SEMARNAT; FAUT 014 to V.H.R.). The study was approved by the International Animal Care and Use Committee (OU IACUC 2023-0247) and the Local Research Ethical Committee of the Instituto de Biología, Universidad Nacional Autónoma de México.

### Snake capture and handling

The study took place from the 5th of June to the 10th of October 2010 between 1000 and 1800 h in Maxtleca de Galeana, Estado de Mexico, in central Mexico (coordinates 19°1′62″, 99°33′49″, 2494 m asl). The test with a human was performed prior to the capture of the snake (see below). We then captured the snakes with a herpetological hook and placed them in a canvas bag, which was carefully closed. Within 1 h, we transported the snakes to a private yard nearby where the other tests took place. The study includes 21 adult snakes: 11 females and 10 males. All captured snakes were included in all tests of this study. Snakes were kept in a concrete enclosure (1.5×1.5×1.2 m), with dry grass as substrate and several stone refuges. Water was provided *ad libitum*. We sexed and then marked the snakes, by stitching two coloured beads at the end of the tail, which is a minimally invasive marking technique (Galdino et al., 2014). After capture, snakes were rested 24 h before the start of the tests. Within 1 week, after all tests were done, the marks were removed, and the snakes were placed in canvas bags and then returned to the location of their capture.

### Tests

We observed and recorded behavioural reactions of the snakes testing the approach of potential predators (i.e. human, bird, or fox), and its behaviour in the presence of a prey (a mouse). We registered essential antipredation behaviours: bite, rattle, and escape, which are considered important in the hierarchical decision making of antipredation behaviour in other snake studies (Gibbons and Dorcas, 2002; Roth and Johnson, 2004). We considered these behaviours essential, because a bite would be dangerous to the potential predator, and the escape will reduce the probability of the encounter, whereas an animal ignorant of the presence of the snake would be notified by the rattle and can respond to avoid a dangerous encounter.

We also recorded body postures: freezing (immobilizing the body), which may avoid detection of the potential predator or prey; dorsoventral compression of the body, a possible threat display in which the snake makes itself appear larger; elevating the head, which suggests that the snake is investigating the animal approaching or getting ready to bite; and hiding the head near or below its body, which may avoid damage to the head in a possible attack (Arnold and Bennett, 1984). Other behaviours observed in some snake species, like the threat behaviour ‘gaping’ (Campbell and Lamar, 2004; Roth and Johnson, 2004; Glaudas and Winne, 2007), erratic movements (used to confuse the predator; Tozetti et al., 2009; Lopes-Assis et al., 2020), approach of the predator (Whitaker and Shine, 1999), or cloacal discharges (Tozetti et al., 2009; Lopes-Assis et al., 2020) were not observed.

We recorded the distance between the predator or prey and the snake at the occurrence of each behaviour, called approach distance. Additionally, we registered flight distance and rattle duration.

Tests were performed on different days at the same time the snakes had been captured, between 11:00 and 18:00 h. The order of the tests was randomized, except for the human test that was always performed before capture. In captivity, all tests with potential predators took place in an area of 2×4 m, with the naturally occurring earth as substrate and this area was divided with string every 10 cm to allow for distance estimations. Tests were performed at natural environmental temperatures (between 10 and 40°C; data in database).

### Human

The approach of a human is often used to illicit an antipredation response in snakes (e.g. Kissner et al., 1997; Whitaker and Shine, 1999; Gibbons and Dorcas, 2002), but here we also wanted to compare the response of the snake to a human with that towards other predatory animals. When a snake was found in the field, a human approached the snake in a direct line at a slow speed of approximately 2 km/h. Another person registered the behaviour of the snake. If the snake escaped, the human kept approaching the snake until 5 min had passed or the approach distance was 0 cm. Locations of the snake and human at the time of the display of the behaviours were noted using small landmarks on the ground and measured with a measuring tape after the capture of the snake. Both persons involved in the tests wore protective clothing, including leather boots and gaiters.

### Terrestrial predator: fox

For the test we selected a fox as a simulated terrestrial predator. We used a life-sized stuffed fox with natural colorations (height of 38 cm) and preheated with sun exposition for snake infrared perception (external temperature of 32°C). The fox was placed on a wooden platform (43×12.5 cm) on wheels. With a system of rope and pulleys the fox was moved in a straight line towards the snake, starting at a distance of 300 cm and reaching the snake in 30 s.

### Aerial predator: overflying and attacking bird

For the tests with a bird as a stimulated arial predator, we used a plastic silhouette of an eagle with a wingspan of 115 cm and a length of 58 cm from head to tail. The bird was moved with a system of rope and pulleys. We performed two trials with the bird. In one test, the bird overpassed the snake in a horizontal plane at an altitude of 3 m; and in another test the bird approached the snake in a dive with a 45° angle, starting at 3 m altitude and 3 m distance from the snake and ending at an altitude of 0 cm at the place where the snake was at the start of the trial. The snake was reached in 30 s. All snakes were tested in both procedures and both tests were compared with the other trials.

### Prey: mouse

The test with the mouse as prey took place in an enclosure of 1.5×1.5×1.2 m, with dry grass as substrate. To allow for the estimation of distances, the area was divided with string in 10×10 cm squares. To stimulate appetite, prior to the trial the snake had been fasted for 5 days (which is likely enough to increase appetite, while still being a short time of fasting for snakes; McCue, 2007). First, snakes were placed in the corner of the enclosure using the herpetological hook, and then an adult mouse was released at the opposite site of the enclosure. Trials lasted 10 min and were recorded with a video recorder (Sony, DSC-S930). 21 mice were obtained from the Faculty of Medicine at the Universidad Nacional Autónoma de México, prior to the tests they were kept in plastic cages, and received rodent feed (Purina) and water *ad libitum*. All mice were eaten, although some after the previous set duration of the trial.

### Data analyses

We tested whether the presence of the behaviours depended on the test (i.e. human, fox, overflying bird, and attacking bird as simulated predator, and mouse as prey) with a binomial general linear mixed model (binomial GLMM), testing for the effect of the test, the type of behaviour, and their interaction, a possible effect of environmental temperature and time the test was performed was also analysed, and identity was included as random factor. Differences in the behaviour first presented during a test was analysed with a multinomial general linear model (mGLM). We also analysed this considering only the essential behaviours (i.e. bite, rattle, and escape). We tested whether the approach distance at which a behaviour was first presented differed among the tests, with GLMM, with separate models for each type of behaviour. GLMMs were also performed to test for differences in reaction distance (approach distance at which the snake first reacted to the predator), flight distance, and rattle duration among tests. When residuals of the model did not have a normal distribution (reaction time, approach distance at which the head was hidden, and rattle duration), models were tested with Gamma distribution, other models were parametric. Additionally, to detect a pattern in behaviours, we formed discrete time Markov chains per test for all behaviours and body postures, and separate Markov chains for only the essential behaviours. All statistics were performed in R (R Core Team, 2019), with the package lme4 for GLMM (Bates et al., 2015), VGAM for multinomial GLM (Yee, 2015), emmeans (Lenth, 2018) and RVAideMemoire (Herve, 2023) for post hoc tests (Lenth, 2018), and markovchain for discrete time Markov chains (Spedicato, 2017).

## Supplementary Material

10.1242/biolopen.061791_sup1 Supplementary information
