# Supplementary material for: Unpredictable aggressive defence of the venomous snake, Crotalus ravus, towards predators and humans
Source: Biol Open. 2025 Apr 2;14(4):bio061791. doi: 10.1242/bio.061791 (PMC11993251; doi:10.1242/bio.061791)
Supplement: Supplementary information [file biolopen-14-061791-s1.pdf]

Table S1. The portion of trials in which the behavior or body posture was presented, was the first behavior showed when all behaviors and body postures were included, was the first essential behavior (i.e., bite, rattle, escape; note that not all snakes displayed these behaviors). n = 21

	Human	Fox	Bird attack	Bird overflying	Mouse
Present					
bite	0.71	0.29	0.29	0.00*	0.57
rattle	0.71	0.52	0.52	0.33	0.43
escape	0.95	0.81	0.62	0.62	0.52
compression	0.19	0.00	0.86	0.95	0.24
freezing	0.38	0.62	0.57	0.57	0.71
hide head	0.00	0.52	0.14	0.05	0.62
elevate head	0.24	0.33	0.19	0.29	0.71
1st behavior					
bite	0.05	0.00	0.00	0.00*	0.00
rattle	0.14	0.14	0.05	0.05	0.14
escape	0.43	0.14	0.14	0.05	0.00
compression	0.00	0.00	0.38	0.33	0.05
freezing	0.29	0.43	0.29	0.33	0.24
hide head	0.00	0.10	0.00	0.00	0.19
elevate head	0.10	0.19	0.14	0.24	0.38
1st essential behavior					
bite	0.24	0.00	0.00	0.00*	0.33
rattle	0.19	0.43	0.43	0.19	0.33
escape	0.57	0.43	0.43	0.52	0.19

* Biting could not occur in the overflying bird test, as the bird was always at a distance of at least 3 m.

Biology Open • Supplementary information

Table S2. Approach distance, flight distance and rattle duration at the first occurrence of the behavior (average +/- sd). Number of snakes that presented the behavior in parenthesis, n = 21. Results of the analyses are also given.

	human	fox	bird attack	bird overflying	mouse	*F*	df	*P*	
Reaction distance	85.67 ± 30.31	124.12 ± 53.66	125 ± 34.26	119.71 ± 54.59	108.14 ± 38.56	4.71	4,98		
Approach distance									
bite	36.87 ± 18.69 (15)	34.33 ± 15.33 (6)	37.17 ± 21.57 (6)	(0)	33.83 ± 17.65 (12)	0.04	3,16	0.99	
rattle	70.27 ± 34.46 (15)	70.68 ± 47.73 (11)	92.82 ± 37.10 (11)	68.71 ± 18.40 (7)	75.00 ± 21.51 (9)	0.88	4,28	0.49	
escape	56.93 ± 31.07 (20)	56.76 ± 21.64 (17)	74.38 ± 42.55 (13)	68.31 ± 26.47 (13)	69.45 ± 34.01 (11)	2.96	1,19	0.10	
compression	21.75 ± 15.54 (4)	(0)	98.92 ± 30.46 (18)	81.40 ± 37.58 (20)	93.80 ± 42.04 (5)	5.79	2,23	0.004	
freezing	114.63 ± 34.62 (8)	112.81 ± 32.50 (13)	110.88 ± 35.49 (12)	102.08 ± 41.14 (12)	100.20 ± 47.12 (15)	0.33	4,35	0.85	
hide head	(0)	99.55 ± 79.25 (11)	71.00 ± 34.51 (3)	9.00 (1)	90.54 ± 32.85 (13)	5.85	3,28		
elevate head	90.40 ± 50.08 (5)	86.14 ± 48.96 (7)	138.50 ± 34.68 (4)	99.67 ± 40.53 (6)	97.97 ± 22.32 (15)	1.35	4,16	0.30	
Flight distance	60.74 ± 32.13 (19)	51.97 ± 19.87 (15)	46.15+20.48 (13)	45.65 ± 19.42 (13)	50.18 ± 21.05 (11)	1.18	1,47	0.33	a
Rattle duration	36.67 ± 28.59 (15)	13.00 ± 10.86 (11)	6.25+3.65 (12)	8.71 ± 6.95 (7)	24.11 ± 13.09 (9)	14.06	4,28		a

a: approach distance did not affect the variable.
Note: The sexes did not differ (P > 0.10). P values are not available for tests with Gamma distribution and are only given for parametric tests.

Biology Open • Supplementary information

Table S3. Tukey post hoc test for the reaction distance.

Test comparation	estimate ± sd	z	P
human - bird attack	-0.41 ± 0.10	-4.04	<0.001
human - bird overflying	-0.34 ± 3.39	-3.39	0.006
human - fox	-0.38 ± 0.10	-3.74	0.001
human - mouse	-0.25 ± 0.10	-2.5	0.09
bird overflying - bird attack	-0.07 ± 0.10	-0.66	0.97
fox - bird attack	-0.03 ± 0.10	-0.31	0.99
fox - bird overflying	0.04 ± 0.10	0.35	0.99
mouse - bird attack	-0.16 ± 0.10	-1.54	0.53
mouse - bird overflying	-0.09 ± 0.10	-0.88	0.90
mouse - fox	-0.13 ± 0.10	1.24	0.73

Table S4. Tukey post hoc test for the approach distance at which compression occurs.

Test comparation	estimate ± sd	z	P
human - bird attack	-18.34 ± 9.53	-4.02	<0.001
human - bird overflying	-50.37 ± 17.01	-2.96	0.015
human - mouse	-65.68 ± 21.13	3.11	0.009
bird overflying - bird attack	-18.34 ± 9.53	-1.92	0.21
mouse - bird attack	-3.03 ± 15.83	-0.19	0.99
mouse - bird overflying	15.31 ± 15.70	0.98	0.75

Biology Open • Supplementary information

Table S5. Tukey post hoc test for rattle duration.

Test comparation	estimate ± sd	z	P
human - bird attack	1.74 ± 0.27	6.53	<0.001
human - bird overflying	1.48 ± 0.30	4.89	<0.001
human - fox	1.15 ± 0.27	4.18	<0.001
human - mouse	0.41 ± 0.28	1.46	0.59
bird overflying - bird attack	0.26 ± 0.32	0.80	0.93
fox - bird attack	0.59 ± 0.30	1.96	0.28
mouse - bird attack	1.33 ± 0.31	4.33	<0.001
fox - bird overflying	0.33 ± 0.32	1.04	0.83
mouse - bird overflying	1.07 ± 0.34	3.17	0.01
mouse - fox	0.74 ± 0.31	2.41	0.11

Biology Open • Supplementary information

Table S6. Markov models for the complete sequence of the behavior of the rattlesnake

<u>Human</u>

behavior \	followed by:							
	Bite	Rattle	Escape	Freezing	Compression	Hide head	Elevate head	None
Bite	0.13	0.30	0.22	0.00	0.04		0.00	0.30
Rattle	0.50	0.00	0.35	0.00	0.00		0.00	0.15
Escape	0.09	0.16	0.22	0.06	0.09		0.06	0.31
Compression	0.50	0.25	0.00	0.00	0.00		0.00	0.25
Freezing	0.13	0.38	0.25	0.00	0.00		0.25	0.00
Hide head								
Elevate head	0.50	0.17	0.33	0.00	0.00		0.00	0.00

<u>Fox</u>

	Bite	Rattle	Escape	Freezing	Compression	Hide head	Elevate head	None
Bite	0.14	0.14	0.00	0.00		0.14	0.14	0.43
Rattle	0.00	0.00	0.64	0.00		0.09	0.09	0.18
Escape	0.22	0.06	0.00	0.28		0.11	0.06	0.28
Compression								
Freezing	0.00	0.13	0.27	0.00		0.47	0.00	0.13
Hide head	0.15	0.23	0.00	0.00		0.00	0.00	0.62
Elevate head	0.00	0.14	0.57	0.14		0.00	0.00	0.14

Bird attack

	Bite	Rattle	Escape	Freezing	Compression	Hide head	Elevate head	None
Bite	0.00	0.00	0.33	0.00	0.00	0.00	0.00	0.67
Rattle	0.14	0.07	0.14	0.07	0.36	0.07	0.00	0.14
Escape	0.00	0.07	0.07	0.27	0.47	0.00	0.00	0.13
Compression	0.08	0.17	0.13	0.13	0.00	0.04	0.00	0.46
Freezing	0.07	0.27	0.27	0.00	0.20	0.07	0.07	0.07
Hide head	0.33	0.33	0.00	0.00	0.00	0.00	0.00	0.33
Elevate head	0.00	0.50	0.00	0.25	0.25	0.00	0.00	0.00

Bird overflying

	Bite	Rattle	Escape	Freezing	Compression	Hide head	Elevate head	None
Bite								
Rattle		0.00	0.29	0.00	0.43	0.00	0.14	0.14
Escape		0.13	0.06	0.25	0.38	0.00	0.00	0.19
Compression		0.11	0.29	0.07	0.00	0.00	0.04	0.50
Freezing		0.07	0.21	0.00	0.57	0.00	0.00	0.14
Hide head		0.00	0.00	0.00	0.00	0.00	0.00	1.00
Elevate head		0.00	0.14	0.14	0.57	0.14	0.00	0.00

Biology Open • Supplementary information

<u>Mouse</u>

	Bite	Rattle	Escape	Freezing	Compression	Hide head	Elevate head	None
Bite	0.11	0.00	0.00	0.17	0.06	0.06	0.33	0.28
Rattle	0.14	0.07	0.43	0.07	0.00	0.07	0.00	0.21
Escape	0.00	0.27	0.00	0.27	0.09	0.09	0.27	0.00
Compression	0.40	0.20	0.00	0.20	0.00	0.00	0.20	0.00
Freezing	0.17	0.00	0.06	0.00	0.00	0.33	0.28	0.17
Hide head	0.13	0.19	0.06	0.06	0.00	0.00	0.25	0.31
Elevate head	0.23	0.10	0.10	0.13	0.06	0.10	0.13	0.16

Table S7. Markov sequence including behaviors in our prediction: freezing, escape, rattle, bite.

Human

behavior \	followed by: Freezing	Escape	Rattle	Bite	None
Freezing	0.00	0.38	0.38	0.25	0.00
Escape	0.06	0.25	0.22	0.16	0.31
Rattle	0.00	0.35	0.00	0.50	0.15
Bite	0.00	0.22	0.30	0.13	0.35

Fox

	Freezing	Escape	Rattle	Bite	None
Freezing	0.00	0.27	0.27	0.07	0.40
Escape	0.28	0.06	0.06	0.28	0.33
Rattle	0.00	0.73	0.00	0.00	0.27
Bite	0.00	0.00	0.14	0.14	0.71

Bird attack

	Freezing	Escape	Rattle	Bite	None
Freezing	0.00	0.33	0.40	0.07	0.20
Escape	0.27	0.07	0.20	0.13	0.33
Rattle	0.07	0.21	0.07	0.21	0.43
Bite	0.00	0.33	0.00	0.00	0.67

Bird overflying

	Freezing	Escape	Rattle	Bite	None
Freezing	0.00	0.36	0.07	0.00	0.57
Escape	0.31	0.13	0.19	0.00	0.38
Rattle	0.14	0.29	0.00	0.00	0.57
Bite					

Mouse

	Freezing	Escape	Rattle	Bite	None
Freezing	0.06	0.17	0.00	0.39	0.39
Escape	0.45	0.00	0.55	0.00	0.00
Rattle	0.07	0.43	0.07	0.14	0.29
Bite	0.22	0.00	0.00	0.22	0.56

Biology Open • Supplementary information

Table S8. The Markov models for the sequence of behavior only including the essential behaviors.

Human

behavior \	followed by: Bite	Rattle	Escape	None
Bite	0.13	0.30	0.22	0.35
Rattle	0.50	0.00	0.35	0.15
Escape	0.16	0.28	0.25	0.31

Fox

	Bite	Rattle	Escape	None
Bite	0.14	0.14	0.00	0.71
Rattle	0.00	0.00	0.73	0.27
Escape	0.33	0.06	0.06	0.56

Bird attack

	Bite	Rattle	Escape	None
Bite	0.00	0.00	0.33	0.67
Rattle	0.21	0.07	0.21	0.50
Escape	0.20	0.27	0.07	0.47

Bird overflying

	Bite	Escape	Rattle	None
Bite				
Rattle		0.29	0.00	0.71
Escape		0.19	0.19	0.63

Mouse

	Bite	Rattle	Escape	None
Bite	0.33	0.00	0.06	0.61
Rattle	0.14	0.07	0.43	0.36
Escape	0.27	0.55	0.00	0.18

Biology Open • Supplementary information
